# Influence of Sleep and Wakefulness States and Physical Activity on Chronic Pain in Temporomandibular Disorders

**DOI:** 10.1111/joor.70076

**Published:** 2025-10-08

**Authors:** Koichiro Uno, Ryota Takaoka, Takafumi Kato, Ayumi Shintani, Shoichi Ishigaki

**Affiliations:** ^1^ Department of Regenerative Prosthodontics Graduate School of Dentistry The University of Osaka Suita Japan; ^2^ Department of Oral Physiology Graduate School of Dentistry The University of Osaka Suita Japan; ^3^ Department of Medical Statistics Osaka Metropolitan University Graduate School of Medicine Osaka Japan

**Keywords:** chronic pain, physical activity, sleep quality, temporomandibular disorders

## Abstract

**Background:**

Chronic pain in patients with temporomandibular disorders is a common and costly social problem. However, studies investigating the direct relationship between sleep and physical activity and pain are insufficient, and the causal relationship between these factors remains unclear.

**Objectives:**

This study aimed to clarify whether sleep conditions and physical activity influence the subjective intensity of chronic pain in patients with temporomandibular disorders.

**Methods:**

The participants consisted of 15 females (mean age: 38.9 ± 10.1 years). Physical activity and total sleep time were evaluated using the Actigraph (AW2, AMI, USA). The recordings were carried out for 14 consecutive days. Pain intensity and subjective sleep quality were also evaluated using the Visual Analogue Scale for 14 consecutive days. The linear mixed‐effects model and Mann–Whitney U‐test were applied for statistical analyses.

**Results:**

The higher the subjective sleep quality the previous night, the lower the jaw pain the next day. The higher the physical activity on the previous day, the lower the jaw pain the next day. The higher the jaw pain on the previous day, the higher the jaw pain the next day.

**Conclusions:**

The subjective intensity of jaw pain was affected by subjective sleep quality, physical activity, and jaw pain the previous day.

## Graphical abstract

The present study suggested that jaw pain decreased when sleep satisfaction of the previous night was high, more physical activity on the previous day was associated with less jaw pain, and the jaw pain on the day of the study was more intense when the jaw pain on the previous day was severe.

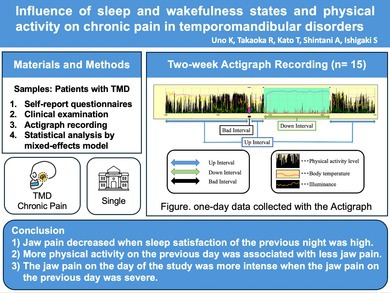

## 1 Background

Pain is an essential sensation for sensing changes in the external and internal environment due to tissue damage [1, 2, 3]. Pain is caused by tissue damage or inflammation due to disease or injury and disappears as healing progresses. However, chronic pain is pain of unknown biological significance that persists beyond the standard healing period of tissue damage [4]. Chronic pain is not responsive to treatment and is considered to be included in the concept of central sensitization syndrome (CSS) [5, 6, 7, 8], a group of disorders proposed by Yunus.

Although pain has traditionally been thought to reduce subjective sleep quality, the hypothesis that sleep disturbance lowers the pain threshold, leading to chronicity and severity of pain, is now supported [9]. Recently, there have been reports that chronic pain is more severe when subjective sleep quality is low or when sleep duration is short [10, 11, 12, 13, 14] and that chronic pain is more severe when physical activity during the day is low [15, 16, 17]. These reports suggest that sleep and physical activity may influence chronic pain. Furthermore, studies targeting fibromyalgia and migraine have reported a relationship between sleep, physical activity, and pain [18, 19, 20], and the necessity of lifestyle improvements as part of rehabilitation for chronic pain is also being discussed. However, studies investigating the direct relationship between sleep and physical activity and pain are still lacking, and the causal relationship between the two remains unclear. Although diurnal variation in chronic pain has been reported [21], there are no reports on the influence of pain on chronic pain over time in patients with temporomandibular disorders (TMD), which are the second most common musculoskeletal pain disorders after chronic low back pain. Chronic pain in patients with TMD is often intractable and represents a major socio‐economic problem that needs to be resolved.

The micro motion logger (Actigraph, AMI Inc., USA), which has a light sensor and an event marker (a button that can be pressed by the user) and uses an inbuilt accelerometer to record movement and proprietary software, is a convenient device for detecting periods of physical activity, rest, and sleep [22]. In validating Actigraph records against polysomnography (PSG), although there are certain limitations in the interpretation of sleep, it has generally been found that they are valid and reliable for assessing sleep in healthy adults with normal sleep cycles [22, 23, 24]. By using Actigraph as a measurement method for sleep and physical activity status, it is possible to extract with a high degree of accuracy the interval of actual sleep during the Down Interval and the number of times of physical activity during the Up Interval, and to obtain more objective data compared to the self‐report measurement.

Therefore, this study aimed to clarify the effects of sleep status, physical activity status, and the previous day's jaw pain on jaw pain in the daily life of patients with TMD by using the micro motion logger longitudinally for 14 consecutive days. We made a null hypothesis that sleep status, physical activity status, and the previous day's jaw pain do not affect jaw pain the next day.

## 2 Methods

### 2.1 Participants

**FIGURE 1:**
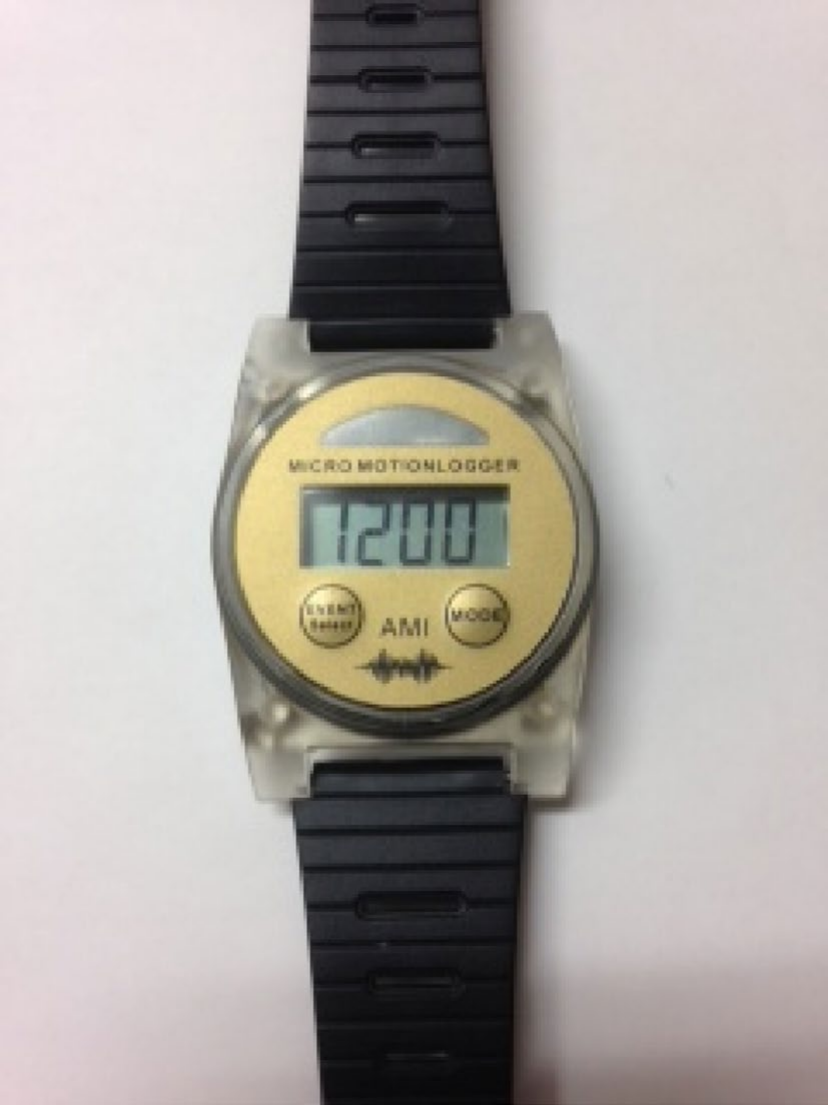
Micromotion logger Actigraph (AW2, AMI, USA).

Sixteen female participants were selected between October 2013 and November 2014 after being briefed about the study, and written consent was obtained. Exclusion criteria were: (1) difficulty in wearing the Actigraph (Figure 1) continuously during the examination; (2) working late‐night shifts [25]; (3) undergoing treatment for psychiatric disorders; (4) pregnant and (5) taking pain medications such as non‐steroidal anti‐inflammatory drugs (NSAIDs), opioids, antiepileptic drugs, antidepressants, steroids. Participants were instructed to use the Actigraph and fill out the questionnaire in advance. One of the 16 participants dropped out of the study because of difficulty in continuing to wear the Actigraph, so the final number of participants was 15 (mean age 38.9 ± 10.1 years). Of the 15 participants, eight were in the Jaw Pain group and had experienced chronic pain in their masticatory muscles for more than three months, and the remaining seven participants were in the No‐Pain group and experienced no pain in their head and neck muscles or temporomandibular joints for the past month. None of the participants complained of joint pain or noises. The maximum assisted opening for all 15 participants was greater than 45 mm. One dentist (SI) conducted a clinical examination based on the Diagnostic Criteria for Temporomandibular Disorders (DC/TMD) and confirmed the diagnosis of TMD by Axis I assessment of DC/TMD [26]. When performing palpation, target pressures are 1 kg to the temporalis and masseter muscles and around the lateral pole of the temporomandibular joint (TMJ) and 0.5 kg to the lateral condylar pole of the TMJ. The palpation calibration was carried out by using a force meter (BASELINE FORCE GUAGE, TRY‐ALL, Japan). MR examinations revealed that, of the patients in the Jaw Pain group, 4 patients had no disc abnormalities; one had disc displacement with reduction in the unilateral TMJ; one had disc displacement with reduction in the bilateral TMJ; and two had disc displacement without reduction in the bilateral TMJ. No erosive degenerative bone change and no moderate or severe joint effusion was observed in TMJ of any participant on MRI. All eight participants in the Jaw Pain group were diagnosed with myalgia but not with althralgia.

### 2.2 Measurement of Total Sleep Time and Physical Activity

**FIGURE 2:**
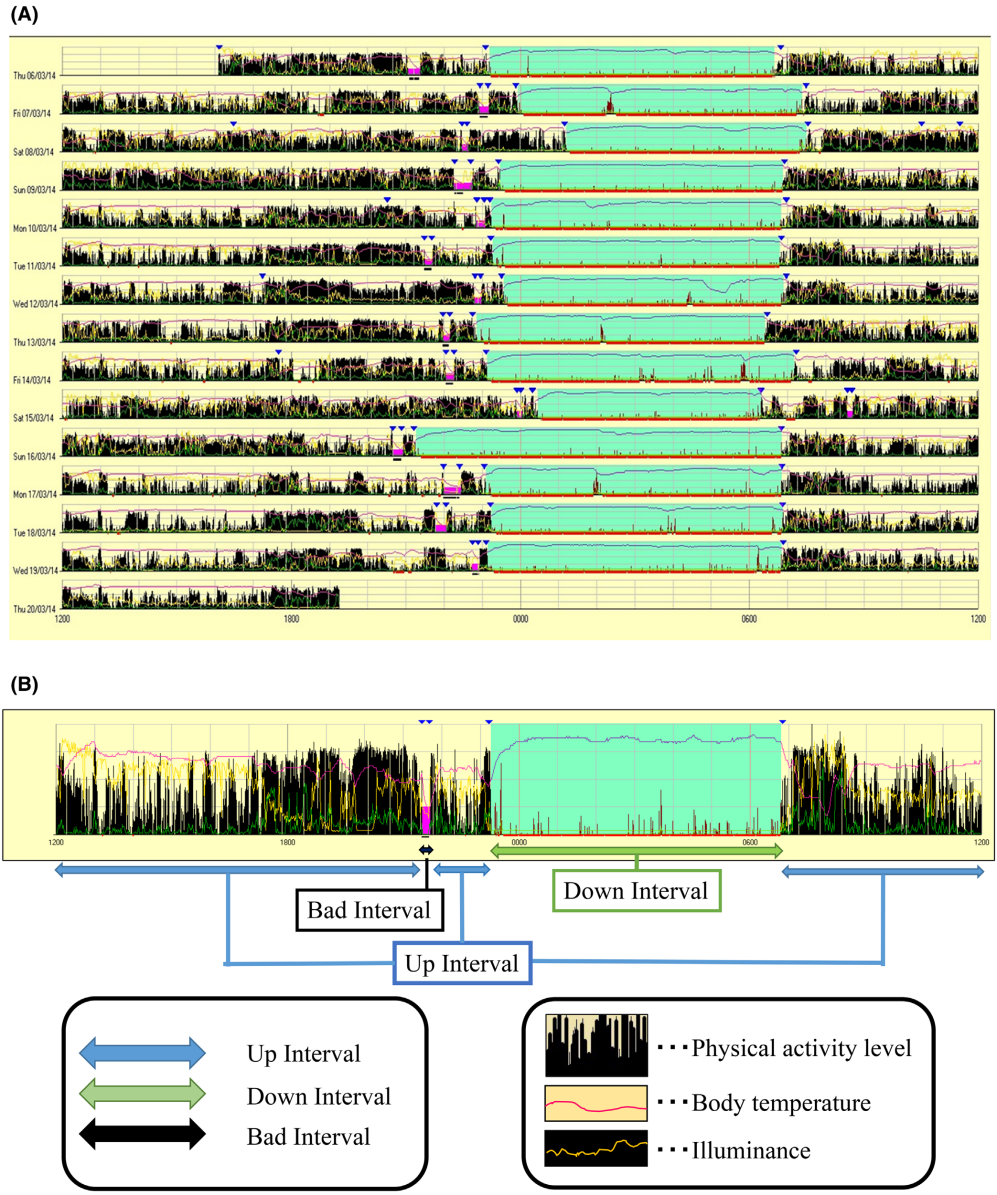
(A) Examples of a single participant's data for 14 consecutive days measured by the Actigraph. (B) Explanation of one night's data by the Actigraph. The black spikes record indicate physical activity levels, the pink wavy lines represent body temperature, and the yellow wavy lines indicate environmental illuminance. Pink intervals are Bad Intervals, which correspond to periods while the Actigraph was removed, such as during bathing. Bad Intervals are set by comparing the time recorded by the participants on the questionnaire with the Actigraph data. The blue‐green section is Down Interval, which is the period from when the participants went to bed until they wake up. The section indicated by the red line at the bottom of the Down Interval is the period that the participants slept. The remaining section is UP Interval, which is the period between waking up and going to bed. This section was used to measure physical activity levels.

Actigraph was used to measure total sleep time and physical activity. The device was worn on the participant's non‐dominant arm and continuously measured day and night for 14 days (Figure 2). The Actigraph is a wristwatch‐type device with a built‐in accelerometer that detects angular acceleration in the *X*‐*Y*‐*Z* directions. Unlike polysomnography, which requires a certain amount of time in a laboratory, the Actigraph can record the participant's daily rhythm, including physical activity and sleep–wake status, making it suitable for measuring biological phenomena in the daily living environment. The ability of the Actigraph to discriminate between sleep and wakefulness has been reported to have an 88%–90% agreement with polysomnography [27, 28, 29].

The Actigraph used in this study was set to 60 s per epoch, and the number of physical activity (unit) indicates the number of times acceleration of 0.01 G or greater was detected during one epoch. The interval from bedtime to waking up was defined as the Down Interval, and the time during the Down Interval when the participant was considered asleep by automatic analysis using the Actigraph was used for analysis as the total sleep time. The median number of physical activities during the Up Interval was used as the number of physical activities during the waking period.

The Actigraph used in this study is waterproof only for daily use, so the participants were instructed to remove the Actigraph when taking a bath. Intervals in which the Actigraph was removed were excluded from the analysis as Bad Intervals.

The Actigraph used in this study was equipped with an “Event” button, which enabled the participant to mark the recorded data by pressing it. In this study, participants were instructed to press the Event button when they went to bed, woke up, removed the Actigraph, and put it back on. Special software (AW2, AMI, USA) was used for the analysis.

### 2.3 Questionnaires

On the first day of the study, the participants completed the following self‐report questionnaires: The Zung Self‐Rating Depression Scale (SDS), the General Health Questionnaire‐28 (GHQ‐28), and the Epworth Sleepiness Scale (ESS).

The visual analog scale (VAS) is widely used to assess pain intensity [30]. Although the obtained values cannot be used to compare pain intensity between patients, they are beneficial for assessing pain trends within individuals [31]. It has been reported that there is diurnal variation in pain, and studies in which pain was measured at two separate times, early in the morning and late at night, have been recognised [21, 32]. In the present study, preliminary experiments showed that TMD patients had less jaw pain until 6 h after waking up and showed increased jaw pain from 6 h after waking up to bedtime and vice versa. Upon waking up daily during the measurement period, participants were asked to complete a questionnaire, where 0 means no pain and 100 means pain as bad as possible. The analysis was performed by dividing each day into two categories. The maximum value of each category for each day was used for analysis.

The VAS was also used to evaluate subjective sleep quality the night before the study [33]. In this study, the participants were asked to fill out the VAS upon waking up, where 0 means the participants were not able to sleep at all and 100 means the participants were able to sleep soundly, and it was used to evaluate the subjective quality of their sleep.

### 2.4 Statistical Analysis

Statistical analysis was performed using IBM SPSS Statistics ver. 22.0 (IBM Japan). Descriptive statistics were used to show the distribution of each covariate. Mann–Whitney's *U* test was used to compare (1) age, (2) ESS, (3) SDS, (4) GHQ‐28, (5) total sleep time, (6) physical activity and (7) the level of sound sleep the previous night between the Jaw Pain group and the No‐Pain group. The significance level was set at 5%. The effects of physical activity and sleep status on jaw pain were adjusted for menstrual period, age, and TMD. Time‐series inconsistent covariates for the dependent variable were excluded, and repeated analysis of 14 consecutive days of data for each participant was required. A mixed‐effects model [34] was used.

In the mixed‐effects model, (1) maximum jaw pain within 6 h after waking; (2) maximum jaw pain after 6 h after waking were used as dependent variables, and (1) total sleep time the previous night; (2) sound sleep level the previous night; (3) the number of physical activities the previous day; (4) maximum jaw pain within 6 h after waking the previous day; (5) maximum jaw pain after 6 h after waking the previous day, were used as covariates. All dependent variables were log‐transformed to normalise the distribution. The odds ratios were calculated. The significance levels were all set at 5%.

All procedures performed in studies involving human participants followed the ethical standards of the institutional and national research committee, and with the 1964 Helsinki declaration and its later amendments or comparable ethical standards. This study was conducted after obtaining approval from the Ethical Review Committee of the Osaka University Graduate School of Dentistry and Osaka University Dental Hospital (approval number: H25‐E9‐2). Informed consent was obtained from all individual participants included in the study. There are no conflicts of interest to report regarding the content of this study.

## 3 Results

### 3.1 Descriptive Statistics

The median age in the Jaw Pain group and the No‐Pain group was 37.5 years old (31.25–41.5 years old in the first and third quartiles) and 38 years old (32–44 years old in the first and third quartiles), respectively. No statistically significant differences were found between the two groups (*p* = 0.867). The median SDS in the Jaw Pain group and the No‐Pain group was 39.5 (37–42 in the first and third quartiles) and 40 (37–44.5 in the first and third quartiles), respectively. There was no significant difference between the two groups (*p* = 0.779). All participants' SDS scores were less than 50, and the scores were within the normal range. The median GHQ‐28 score in the Jaw Pain group and the No‐Pain group was 8.5 (6.25–10.75 in the first and third quartiles) and 1 (0–2 in the first and third quartiles), respectively. There was a significant difference between the two groups (*p* < 0.001). The median ESS in the Jaw Pain group and the No‐Pain group was 11.5 (7.5–14.5 in the first and third quartiles) and 5 (4–12 in the first and third quartiles), respectively. There was no significant difference between the two groups (*p* = 0.152).

**FIGURE 3:**
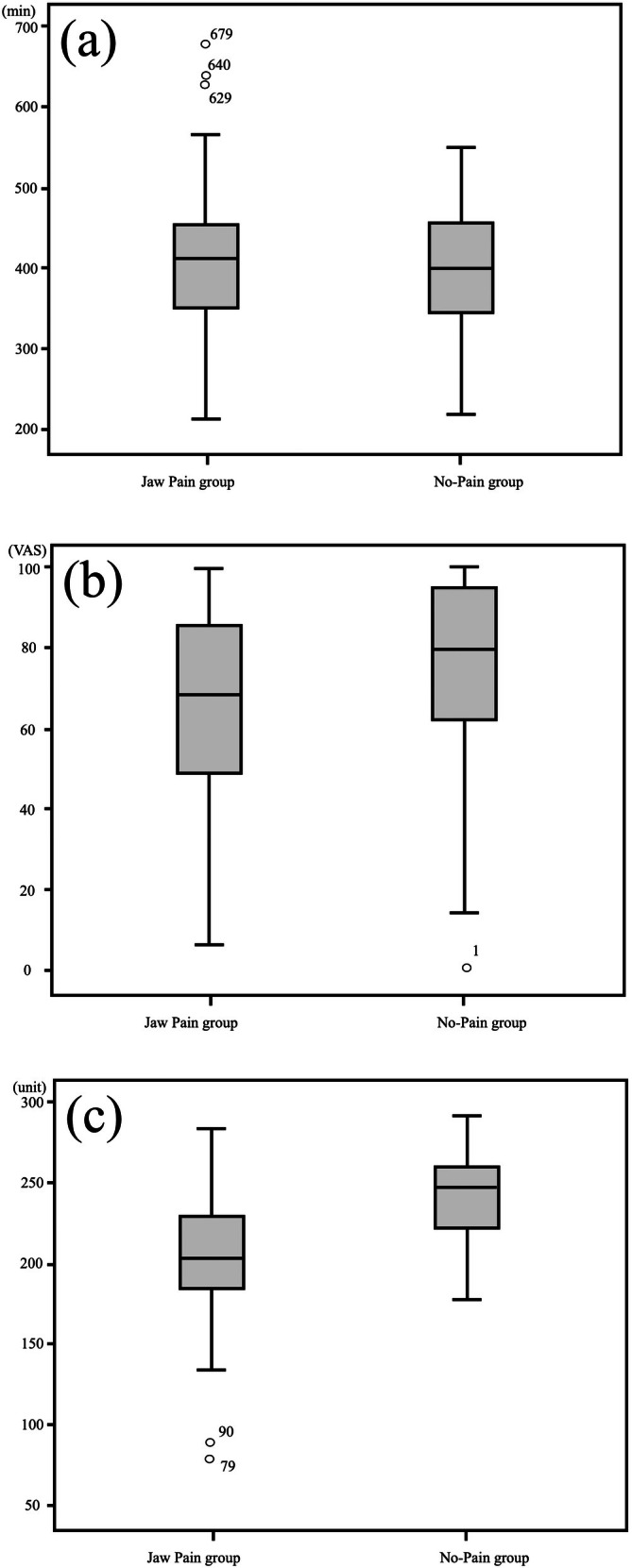
Distribution of total sleep time (a), subjective sleep quality on the previous day (b), and physical activity (c).

Comparison between the Jaw Pain group and No‐Pain group showed no significant difference in total sleep time (*p* = 0.726). The median total sleep time was 400.50 min (345.75–454.75 in the first and third quartiles) in the No‐Pain group and 413.00 min (350.00–455.75 in the first and third quartiles) in the Jaw Pain group (Figure 3). However, both the level of sound sleep the night before (*p* = 0.009) and the number of physical activities (*p* < 0.001) were significantly lower in the Jaw Pain group than in the No‐Pain group. The median number of minutes of sound sleep the previous night was 80.00 (60.25–95.50 in the 1st–3rd quartiles) in the No‐Pain group and 68.50 (48.25–86.00 in the 1st–3rd quartiles) in the Jaw Pain group. The median number of physical activities was 247.50 units in the No‐Pain group (222.62–259.75 in the 1st–3rd quartile) and 203.00 units in the Jaw Pain group (184.25–230.25 in the 1st–3rd quartile). No comparison was made for the pain covariate since the No‐Pain group had a value of 0.

### 3.2 Influence of the Previous Night's Sleep on Jaw Pain (Table 1)

**TABLE 1:** The results of the mixed‐effect model.

Covariate variables	Dependent variables							
	The maximum jaw pain in the first period				The maximum jaw pain in the second period			
	Odds ratio	*p*	95% CI		Odds ratio	*p*	95% CI	
			Min	Max			Min	Max
Total sleep time last night (min)	0.999	0.215	0.998	1.000	0.999	0.230	0.998	1.000
Subjective assessment of the quality of sleep last night^a^	0.991	0.000	0.988	0.995	0.993	0.000	0.989	0.996
The median number of the physical activity on the previous day (unit)	0.997	0.045	0.994	1.000	0.997	0.038	0.994	0.999
The maximum jaw pain in the first period on the previous day^a^	1.009	0.012	1.002	1.017	1.003	0.325	0.996	1.011
The maximum jaw pain in the second period on the previous day^a^	1.006	0.042	1.000	1.013	1.004	0.143	0.998	1.011

^a^ 0–100 mm visual analogue scale.

A significant relationship was found between the level of sound sleep the night before and the maximum value of jaw pain within 6 h after waking, indicating that the higher the level of sound sleep the night before, the weaker the jaw pain within 6 h after waking (odds ratio: 0.991, *p*‐value: 0.000, 95% CI: 0.988 to 0.995). A significant relationship was also observed between the degree of sound sleep the night before and the maximum value of jaw pain 6 h after waking, indicating that the higher the degree of sound sleep the night before, the weaker the jaw pain 6 h after waking (odds ratio: 0.993, *p*‐value: 0.000, 95% CI: 0.989 to 0.996).

On the other hand, there was no significant relationship between total sleep time of the previous night and maximum jaw pain within 6 h after waking (odds ratio: 0.999, *p*‐value: 0.215, 95% CI: 0.998–1.000). Similarly, there was no significant relationship between total sleep time the night before and maximum jaw pain 6 h after waking (odds ratio: 0.999, *p*‐value: 0.230, 95% CI: 0.998–1.000).

### 3.3 Influence of Physical Activity on the Previous Day on Jaw Pain (Table 1)

A significant relationship was found between the number of physical activities on the previous day and the maximum value of jaw pain within 6 h after waking, indicating a higher number of physical activities on the previous day. The weaker the jaw pain within 6 h after waking (odds ratio: 0.997, *p*‐value: 0.045, 95% CI: 0.994 to 1.000). A significant relationship was also found between the number of physical activities on the previous day and the maximum value of jaw pain 6 h after waking, indicating the higher number of physical activities on the previous day (odds ratio: 0.997, *p*‐value: 0.038, 95% CI: 0.994–0.999).

### 3.4 Influence of Jaw Pain on the Previous Day on Jaw Pain (Table 1)

A significant relationship was found between the maximum jaw pain within 6 h of waking up the previous day and the maximum jaw pain within 6 h after waking up the next day. This result indicates that the stronger the jaw pain within 6 h after waking up on the previous day, the stronger the jaw pain within 6 h after waking up on the next day (odds ratio: 1.009, *p*‐value: 0.012, 95% CI: 1.002 to 1.017). Similarly, a significant relationship was found between the maximum jaw pain 6 h after waking the previous day and the maximum jaw pain within 6 h after waking on the same day. This finding indicates that the more severe the jaw pain after 6 h after waking on the previous day, the stronger the jaw pain within 6 h after waking on the same day (odds ratio: 1.006, *p*‐value: 0.042, 95% CI: 1.000–1.013).

On the other hand, there was no significant relationship between the maximum value of jaw pain within 6 h after waking on the previous day and jaw pain after 6 h after waking (odds ratio: 1.003, *p*‐value: 0.325, 95% CI: 0.996–1.011). Similarly, there was no significant relationship between maximum jaw pain 6 h after waking and maximum jaw pain 6 h after waking on the previous day (odds ratio: 1.004, *p*‐value: 0.143, 95% CI: 0.998–1.011).

## 4 Discussion

The incidence of chronic pain disorders such as TMD is higher in women than in men [35, 36, 37, 38, 39, 40, 41, 42, 43, 44, 45]. So we limited the number of participants to women in this study. In addition, there was a report that men and women perceive pain in the facial area differently [46]. Furthermore, women's pain sensitivity differs from men's due to the effects of changes in hormonal balance, such as changes in blood levels of estrogen [47, 48], so there may be problems in conducting the analysis together with men. In addition, statistical processing in this study requires correction for menstruation, age, and TMD. In addition, those who worked late‐night shifts were excluded because they were reported to be more prone to insomnia [25].

The accuracy of Actigraph in determining sleep and wakefulness has been reported to have a high correlation of 88% to 90% with polysomnography [27, 28, 29]. However, although the Actigraph can determine sleep and wakefulness, it is difficult to determine the depth of sleep and discriminate factors that may reduce subjective sleep quality, such as sleep apnea, snoring, and sleep bruxism. In other words, evaluating the quantity of sleep is possible, but evaluating the quality of sleep is difficult. For this reason, in this study, only total sleep time was determined by the Actigraph as an objective outcome of sleep status. In contrast, the participant's self‐report, in which the participant's sleep satisfaction was recorded on the VAS, was simultaneously recorded as a subjective outcome of sleep status.

The mean or median of the number of physical activities calculated by the Actigraph has been reported [49, 50, 51]. The AW2 software used in this analysis can evaluate the median or mean value of the number of physical activities measured during the daily Up Interval as the participant's physical activity. In this study, observation of the data automatically recorded by the Actigraph revealed that the number of physical activities was extremely high in some areas and low in others. The use of the mean value was likely to be affected by these extreme data. For these reasons, the median of physical activities during the Up Interval of the day was used as the outcome of the physical activity status measurement in this study.

The mixed‐effects model used in this study includes fixed and random effects and is a statistical method suitable for analysing continuous data [34]. It can model inter‐participant variability and conduct analyses focused on intra‐participant variability [34]. In this study, statistical analysis was conducted to examine the effects of participants' sleep and physical activity status on jaw pain. Typically, when observing the effects of covariates on dependent variables across participants, it is necessary to conduct separate analyses for participants with pain and without pain. However, the statistical analysis method used in this study allows us to exclude inter‐participant variability, observe within‐participant variability, and correct for the effects of various pain confounders, making it possible to analyse participants without pain with jaw pain simultaneously. Rather than comparing participants with and without jaw pain, a repeat analysis was conducted to observe the effect of covariates such as sleep duration and physical activity on the variation in jaw pain over 14 days for a single participant. The 14‐day data for 15 participants were then corrected for pain confounders such as menstrual period, age, and presence or absence of TMD, and the overall trend was analysed. The results were completely valid, but it was essential to correct the TMD status when analysing the mixed‐effects model because the number of sleep soundly and the number of physical activities the night before were significantly lower in the Jaw Pain group compared to those in the No‐Pain group.

In addition, most of the previous studies that explored the relationship between sleep and physical activity status and pain were cross‐sectional studies [10, 12, 13, 14] or review articles [15, 16], and even if a significant relationship was found, the causal relationship could not be mentioned. In contrast, the present study was a prospective longitudinal study with 14 consecutive days of measurements, and all covariates occurred before the results, which we consider to provide results that could be referred to as a causal relationship.

The inferior quality of sleep in patients with chronic pain compared to those without chronic pain has been repeatedly confirmed in clinical studies of many chronic pain disorders, including fibromyalgia [11, 14], low back pain [10, 11], and temporomandibular joint disorder [9, 52]. However, since these clinical studies were cross‐sectional, it was impossible to clarify whether sleep is disturbed by chronic pain, whether poor subjective sleep quality is one of the risk factors for chronic pain, or whether both are coexistent. The results of this study indicate that jaw pain is significantly weaker when the level of sound sleep the night before was higher. In other words, a decrease in the level of sound sleep may lead to increased jaw pain. For example, the results using the maximum value of jaw pain within 6 h after waking as the dependent variable showed that the first quartile of the previous night's sound sleep level was 48.25 in participants in the Jaw Pain group. Assuming that the sound sleep level increased to a median of 68.50, the jaw pain was 0.83 times greater than the previous night's sound sleep level. Assuming that the median level of sound sleep increased to 68.50, jaw pain decreased 0.83‐fold (odds ratio: 0.991). Furthermore, judging from the odds ratio, the difference between the effect of the level of sound sleep the night before on jaw pain within 6 h after waking and the effect of the level of sound sleep after 6 h after waking decreased. This suggests that the level of sound sleep has an equal effect on the degree of jaw pain the following day.

Davin et al. reported that a longer total sleep time decreased jaw pain the next day, which differs from this study [11]. However, Lunde et al. compared the total sleep time of a group of chronic pain patients and a control group with no complaints of poor sleep using an Actigraph. They reported no significant difference between them [10]. Since the Actigraph does not measure sleep depth, the sleep time is almost the same for participants who sleep for long periods but do not get deep sleep due to awakenings and those who sleep for long periods without awakenings. On the other hand, the measurement of the level of sound sleep the night before by the questionnaire (VAS) is based on subjectivity and thus is considered to reflect the depth of sleep more than the length of sleep itself. This reason may be why the degree of sound sleep the night before significantly affected jaw pain in this study, while total sleep time had no significant effect. This study indicates that poor sleep status, or low subjective satisfaction with the previous night's sleep upon awakening, may aggravate chronic jaw pain.

This study conducted a longitudinal study of the effect of the previous day's physical activity level on chronic pain by making continuous measurements over 14 days. The results of this study indicated that jaw pain tended to be weaker when the amount of physical activity on the previous day was higher. The first quartile of physical activity in participants with jaw pain was 184.25 units, but when this quartile rose to a median of 203.00 units, jaw pain decreased approximately 0.94‐fold (odds ratio: 0.997). Furthermore, the odds ratios of the effect of the number of physical activities on jaw pain within 6 h after waking and the effect of the number of physical activities on jaw pain after 6 h after waking were both 0.997. This result suggests that the number of physical activities on the previous day affects the degree of jaw pain equally throughout the day, as does the degree of sound sleep the previous night.

This study has several limitations. First, one examiner confirmed the diagnosis of TMD, and having one examiner without calibration increases the risk of diagnostic bias. Second, Actigraph only measures sleep duration and wakefulness, and does not capture sleep stages (REM, N1, N2, N3), sleep apnea, and sleep bruxism. Additionally, the median number of physical activities measured by actigraphy was used for statistical analysis in this study. However, further investigation is needed for other measurement variants such as the intensity of physical activity. The third limitation of the study was that daily maximum jaw pain was assessed solely via self‐reported VAS. To comprehend the characteristics of chronic pain, other pain scales such as average pain, pain frequency, pain duration, functional impairment, and emotional impact should be evaluated. The fourth limitation of the study was that the subjects in this study were a small sample of Asian females with masticatory muscle pain, and the results of this study may not be applicable to all patients with chronic pain. In addition, stress and lifestyle factors may influence pain sensitivity, but this study could not eliminate their effects. Thus, further experiments with a larger sample that take into account race, gender, and subtypes of TMDs (myogenous, arthrogenous, disc displacement, and so on) will be necessary in the future.

Within the limitations of the study, the results may be applicable to patient education and rehabilitation programs. In particular, since sleep and activity on the previous day may affect pain on the following day, recommending appropriate sleep to patients and encouraging those with chronic myalgia to exercise or go for walks could reduce chronic pain.

## 5 Conclusion

Sleep/wakefulness, physical activity, and pain level were measured for 14 consecutive days using an Actigraph and self‐report. The data obtained were statistically analysed using a mixed‐effects model to search for risk factors affecting jaw pain, with the following results.

1. Jaw pain decreased when sleep satisfaction from the previous night was high.
2. More physical activity on the previous day was associated with less jaw pain.
3. The jaw pain on the day of the study was more intense when the jaw pain on the previous day was severe.

These results suggest that poor sleep and physical inactivity may be risk factors for chronic jaw pain.

These findings clarify the influence of sleep and physical activity status on jaw pain and indicate the clinical usefulness of understanding patients' sleep and physical activity status and correcting poor sleep and poor physical activity for individualised treatment of patients with chronic pain in the jaw. However, these results are limited to a small group of patients with chronic myalgia. Further research will be required in a wider age range, including males and patients with temporomandibular joint pain.

## Author Contributions

**Koichiro Uno:** visualisation, data curation, formal analysis, writing – original draft. **Ryota Takaoka:** supervision, conceptualisation, funding acquisition, writing – review and editing. **Takafumi Kato:** validation, conceptualisation, writing – review and editing. **Ayumi Shintani:** formal analysis, writing – review and editing. **Shoichi Ishigaki:** project administration, validation, conceptualisation, funding acquisition, writing – review and editing.

## Conflicts of Interest

The authors declare no conflicts of interest.

## Data Availability

The datasets used and/or analysed during the current study are available from the corresponding author on reasonable request.
